# Downbeat Nystagmus: Case Report, Updated Review, Therapeutics, and Neurorehabilitation

**DOI:** 10.3390/brainsci15080859

**Published:** 2025-08-13

**Authors:** T. Maxwell Parker, Ruben Jauregui, Scott N. Grossman, Steven L. Galetta

**Affiliations:** 1 Department of Neurology, New York University Grossman School of Medicine, 222 E 41st St., 14th Floor, New York, NY 10017, USA; rjaureg91@gmail.com (R.J.);; 2 Department of Ophthalmology, New York University Grossman School of Medicine, New York, NY 10017, USA

**Keywords:** downbeat nystagmus, nystagmus, rehabilitation, neuro-rehabilitation, vestibular rehabilitation neuro-otology, vestibular neurology, central vestibular disorders, neuro-ophthalmology

## Abstract

**Introduction**: Downbeat nystagmus (DBN) is an ocular motor disorder characterized by persistent to-and-fro eye movements with a slow phase directed upwards and a corrective fast phase downwards. DBN in the context of myelin oligodendrocyte glycoprotein-associated disorder (MOGAD) represents a rare clinical presentation. **Case Presentation**: A 24-year-old male with MOGAD presented with DBN, status epilepticus, and longitudinally extensive transverse myelitis (LETM). **Intervention**: The clinical course, diagnostic findings, and management approach are described in detail within the full report. **Outcomes**: The patient at follow-up was able to ambulate independently, and his nystagmus had improved. He continued to demonstrate transient DBN on supine positioning and head-shaking test. **Conclusions**: This case report contributes to the understanding of DBN as a manifestation of MOGAD. The accompanying literature review examines the neuroanatomy, pathophysiology, and emerging therapeutic approaches for DBN, providing context for this unusual presentation.

## 1. Introduction

Downbeat nystagmus (DBN) is an ocular motor disorder commonly encountered by the neurologist or ophthalmologist [1]. DBN is characterized by slow upwards drift (slow phase) and fast phases directed downwards (fast phase). DBN has been demonstrated to be caused by a large variety of pathologies affecting both central and peripheral arcs of the vestibular-ocular control. Although the exact incidence remains unknown, in a large cohort of 3471 patients presenting to a tertiary vestibular center, it was found that 54 individuals had DBN (1.6%) [2]. Central causes, which may account for approximately two-thirds of all cases of DBN, include hyperacute disorders such as stroke or seizure, subacute conditions like compressive lesions or paraneoplastic disorders, or slowly progressive metabolic disorders such as thiamine or B12 deficiency [3]. Peripheral disorders (e.g., Meniere’s Disease, anterior canal Benign Paroxysmal Positional Vertigo (BPPV), labyrinthine trauma) can manifest on examination with DBN or mixed semiologies of nystagmus in various circumstances and are thus an important consideration in the differential diagnosis [4,5,6]. The symptoms experienced by those with both peripheral and central causes of DBN can include oscillopsia, vertigo, disequilibrium, motion sickness, or gait instability [1]. DBN may also be incidentally discovered in an asymptomatic individual, and infrequent beats can be seen in the general population [7]. This patient report describes a 24-year-old male with MOG-associated disorder (MOGAD) presenting with status epilepticus and longitudinally extensive transverse myelitis (LETM) involving the cervico-medullary junction. DBN was observed on examination. We discuss the clinical course, diagnostic findings, and management, followed by a review of the neuroanatomy, pathophysiology, and therapeutic options for DBN.

## 2. Case Presentation

**Figure 1:**
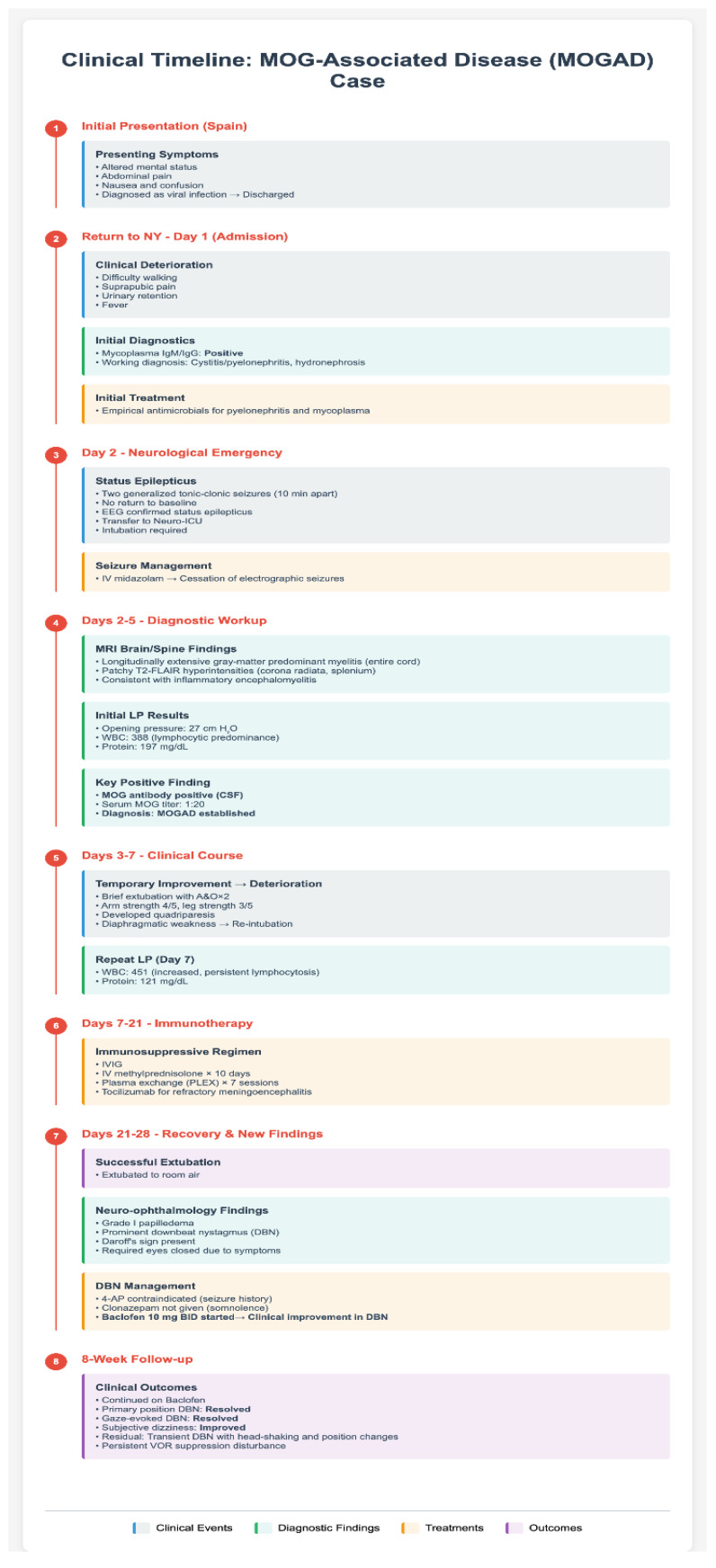
Flowchart depicting the patient’s clinical course, including events, diagnostic results, treatments, and outcome.

A 24-year-old male with no medical history presented with altered mental status (AMS) after a recent trip to Spain, where he experienced abdominal pain, nausea, and confusion. Initial evaluation at an emergency department in Spain suggested a viral infection, and he was subsequently discharged. Upon returning to New York, he developed difficulty walking, suprapubic pain, and urinary retention. He was admitted for cystitis/pyelonephritis, hydronephrosis, and fever. Mycoplasma IgM/IgG was positive, prompting empirical antimicrobial therapy to cover pyelonephritis and mycoplasma infection. See Figure 1 for timeline and review of clinical course. In keeping with case report guidelines, a completed CARE checklist can be found in the Supplementary Material.

On the second hospital day, he experienced two generalized tonic-clonic seizures (GTCs) separated by 10 min, without return to baseline, confirmed as status epilepticus via EEG. He was transferred to the neuro-intensive care unit (ICU) for management. Due to on-going seizure activity, he was intubated. EEG later confirmed cessation of electrographic seizure after addition of intravenous midazolam.

**Figure 2:**
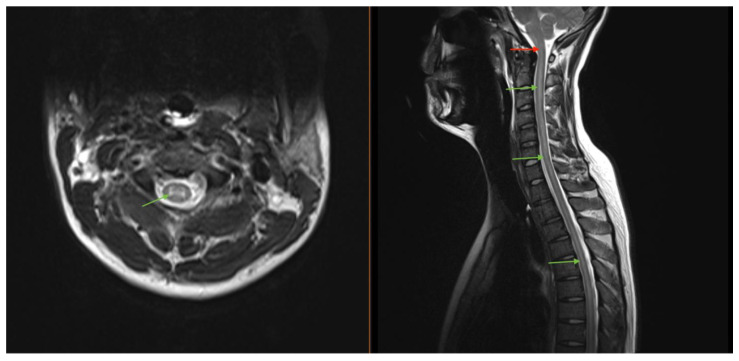
MRI imaging of our patient who was found to have a longitudinally extensive gray matter lesion (green arrows) extending cranio-caudally from the brainstem through the lumbar spinal cord. The lesion involvement of the dorsal medulla (red arrow) was thought to represent the pathologic etiology of the DBN seen in our case.

Magnetic resonance imaging (MRI) of the brain/total spine revealed longitudinally extensive gray-matter predominant myelitis of the entire spinal cord (Figure 2) and patchy T2-FLAIR hyperintensities in the brain (corona radiata and splenium), suggestive of inflammatory encephalomyelitis. Lumbar puncture (LP) showed an opening pressure of 27 cm H_2_O, 388 nucleated cells with lymphocytic predominance, and a protein 197 mg/dL. Several viral and autoimmune panels (including Aquaporin-4, herpes, and paraneoplastic antibodies) were negative in the cerebrospinal fluid (CSF), except for a positive CSF myelin oligodendrocyte glycoprotein (MOG) antibody titer that was performed on a research basis. A corresponding MOG panel in the serum resulted at 1:20. The diagnosis of MOG-associated disease (MOGAD) was made.

The patient was briefly extubated, with brief recovery of alertness and orientation to person and place (A&O × 2), 4/5 strength in arms, and 3/5 in legs. However, he developed worsening quadriparesis and diaphragmatic weakness, necessitating re-intubation. A repeat LP five days later showed nucleated cells increasing to 451 with persistent lymphocytic predominance and elevated protein to 121 mg/dL. Treatment included intravenous immunoglobulin (IVIG), a 10-day course of intravenous methylprednisolone (IVMP), and plasma exchange (PLEX) for seven planned sessions. Tocilizumab was administered for refractory meningoencephalitis and was well-tolerated.

The patient was eventually extubated to room air. At this time, neuroinflammatory and ophthalmology evaluations noted grade I papilledema and prominent DBN requiring the patient to keep his eyes closed. The DBN was evident in primary gaze but worsened in extremes of lateral gaze (see Supplementary Video S1). In horizontal gaze, it developed an admixed horizontal component with down-left beat in left gaze and down-right beat in right gaze, overall, consistent with Daroff’s sign [3]. Due to the history of status epilepticus, 4-aminopyridine (4-AP) was not utilized. Baclofen and clonazepam were both considered for DBN management, but ultimately clonazepam was rejected due to the patient’s somnolence. Baclofen was initiated and maintained at a dosage of 10 mg twice daily, yielding clinically observable improvements in amplitude and frequency of the DBN on subsequent examinations with minimal effects on sedation. The patient was deemed medically stable for rehabilitation with improvement in subjective dizziness and downbeat nystagmus. At follow-up 8 weeks later, he had continued on Baclofen and his primary position and gaze-evoked DBN had resolved with further concomitant symptom improvement. Vertigo and DBN, however, could be transiently evoked via head-shaking and when transitioning from sitting to supine, along with a lingering disturbance of VOR suppression.

## 3. Clinical Neuroanatomy and Assessment of Downbeat Nystagmus

The anatomy of the vertical Vestibulo-Ocular Reflex (VOR) differs from its horizontal counter-part [7]. A crucial key point in the pathophysiologic emergence of downbeat nystagmus is to conceptualize the natural asymmetry in the upwards and downwards reflexes to vertical head movement [3,7,8]. For downward head movements, the semicircular canals relay a stronger stimulus through the afferent arc of the vertical VOR than for upward head movements. This is because the anterior semicircular canal (ASC), responsible for the VOR in response to downwards head movements, is more in line with the vertical rotational plane than is the posterior semicircular canal (PSC)—which is responsible for the VOR from upward head movements [3,7]. This is also reflected in the physiological “time constant”, which is defined as the time over which the nervous system will decay (and adapt to) a velocity stimulus exerted on the vestibular organ [9]. For downward head movements, the time constant is 15 s, while for upward head movements, the time constant is 8 s [8].

Clinically, it is critical to assess whether downbeat nystagmus is present spontaneously in the primary position independent of head position (supine, prone, upright), present spontaneously in primary position dependent to head position, or whether it only emerges in an eccentric position (gaze-evoked nystagmus) [3,7]. The distinction allows for localization of the dysfunctional circuit, thus narrowing or even solidifying the diagnosis of the primary pathology in question.

### 3.1. Gravity-Independent Spontaneous Downbeat Nystagmus

Although the precise circuitry regarding the emergence of spontaneous gravity-independent DBN in pathology remains uncertain, it is likely that multiple physiologic features contribute [7]. The flocculus/paraflocculus complex possesses disproportionate inhibitory output on the tonic input of the ASC as compared to the PSC. If this inhibition by the flocculus were to be released, the eyes would drift upwards (slow phase) as if our head was rotating downwards, and a correctional downwards saccade (fast phase) is necessary.

### 3.2. Gravity-Dependent Spontaneous Downbeat Nystagmus

DBN that emerges only in various changes in head positions is considered a *gravity-dependent* DBN [7]. The circuit centers around the otolith organs (the macule of the saccula and utricle of the inner ear) and their projections in response to the changes in sensation of gravity’s force as the head moves [10,11,12]. In the upright position, the disproportionate stimulus from the ASC is generally sufficient to counter-balance the downward effect of gravity. However, if the head orientation is changed to supine or prone, there must be an appropriate adjustment to the tonic imbalance from the ASC in comparison to the PSC. The nodulus and ventral uvula of the cerebellum have been found to integrate the dynamic inputs from the otolith organs to create an output that stabilizes the visuo-vestibular interaction as the head changes orientation [10,13]. If the nodulus or uvula were to be lesioned, changes in gravitational sensation would not allow for an appropriate counter-balancing force to stabilize the eyes, leading to DBN in the prone position and often a UBN in the supine position [14,15].

### 3.3. Gaze-Evoked Downbeat Nystagmus

When DBN is only evoked upon eccentric gaze, it is termed gaze-evoked nystagmus, and it can typically be identified as a malfunction of the vertical neural integrator (VNI) [16]. Neural integration occurs as part of the “pulse-step” formula of saccades needed to accurately overcome orbital forces driving the eye towards primary gaze to land the fovea on target, as well as to keep it there [7,17]. Gaze-evoked vertical nystagmus occurs as a part of dysfunction of the neural integrator in the “step” component [7,18]. Specifically, there is a breakdown of the continuous integration of the forces needed to counter-balance the natural resistive forces of the orbit in a specific position, which aim to pull the pupil to the primary position [7,17,18]. When this breaks down, the forces of the orbit overcome the inaccurate or insufficient counter-balancing by the vertical integrator, and the eyes drift briefly back towards the primary position (the slow phase) before being re-routed back to the intended position of gaze by a saccade (the fast phase). This nystagmus will therefore be named for the direction of the eccentric gaze. The site where the forces are integrated to hold the eye in a vertical eccentric position after a saccade is the interstitial nucleus of Cajal (INC) in the mesencephalon [16,18]. It follows that any injury to the INC or its afferent or efferent projections can manifest in a gaze-evoked nystagmus in the vertical plane. Importantly, the paramedian tracts from the vestibular nuclei coursing through the lower brainstem to the INC or the posterior commissure are possible sites of lesions in the brainstem to cause gaze-evoked DBN [19,20].

## 4. Pharmacologic Therapy for Downbeat Nystagmus

Therapeutic options for downbeat nystagmus are geared towards a multi-disciplinary approach centered around pharmacological interventions (Table 1), visuo-vestibular rehabilitation, optical interventions (i.e., prisms), and, if possible, correction of the underlying cause.

### 4.1. Aminopyridines

In terms of pharmacologic medications, consideration should first be given to whether the patient is a candidate for dalfampridine, which is the sustained-release formulation of 4-aminopyridine (4-AP) [21]. 4-AP is a voltage-gated potassium channel blocker that has been shown to restore the pacemaker potential of inhibitory cerebellar Purkinje cells via prolonging action-potential length and increasing hyperpolarization amplitudes to near physiologic range [22,23]. Indeed, 4-AP has been shown in two seminal randomized controlled trials to reduce slow phase velocities of DBN [21,24]. In the RCT from Claassen et al., improvements were also noticed in visual acuity, postural sway, and measures of gait and stability [21]. Multiple series and non-randomized controlled trials have demonstrated similar reductions in slow phase velocity (SPV), along with varying degrees of improvement in gait and symptom relief [21,25,26,27,28]. The recommended dosage for dalfampridine is 10 mg twice daily [21]. Contraindications to dalfampridine include patients with a history of seizures (as in our patient) and those with a creatinine clearance of 50 mL/min or less [29]. In the United States, dalfampridine is approved for use in gait disturbance in multiple sclerosis but not currently for downbeat nystagmus.

3,4-diaminopyridine (3,4-DAP), prescribed as amifampridine, is closely related biochemically to 4-AP and has similarly been used with success in DBN [24,30,31]. Methylation at the third carbon is responsible for its pharmacokinetic properties and renders it a less lipid soluble, which decreases its ability to cross the blood–brain barrier compared to 4-AP [32]. Because of decreased central nervous system penetration, patients receiving 3,4-DAP in a head-to-head RCT demonstrated an inferior, but still significant, decrease in SPV and symptoms compared with 4-AP [24]. Nevertheless, 3,4-DAP in a sustained release form, amifampridine, could be an excellent alternative for DBN in patients with renal impairment, in which 3,4-DAP has no contraindication [33]. Amifampridine (i.e., Firdapse^®^), like 4-AP, may be associated with seizures, occurring at a rate of 2% [33]. Seizures may particularly occur in those who have co-morbid medical conditions or medications that lower the seizure threshold, including intracerebral neoplasms and theophylline [34,35,36].

### 4.2. Clonazepam

Evidence for clonazepam derives from two case series totaling 17 patients where 12 out of 17 patients with DBN showed a robust response [37,38]. Clonazepam dosing has found efficacy starting at low doses of 0.5 mg twice daily, with up titration to 1 mg twice daily as tolerated. Clonazepam has superior tolerability as compared to other benzodiazepines but still suffers from the drawbacks of sedation, potential for dependence, and less accessibility given its controlled substance regulation in the US.

### 4.3. Baclofen

Baclofen has also been shown in two series of 17 patients which improved slow phase velocities and subjective vertiginous symptoms [39,40]. The mechanism of action of Baclofen is the generalized restoration of GABAergic inhibition by the flocculo-nodular structures onto the anterior semicircular canal’s projections to the vestibular nuclei [40]. However, not all patients are responders to baclofen. Effective regimens vary, but generally starting dosages at 5 mg three times daily has been effective, with room for adjustment in dosage strength and timing [40].

### 4.4. Gabapentin

One RCT study demonstrated minimal effects on downbeat nystagmus in 6 patients but a robust response for pendular nystagmus [41]. The authors concluded that only occasional patients with DBN may benefit from Gabapentin, and the reason behind this remains unknown. However, a survey of UK ophthalmologists and neurologists self-reported gabapentin to be an effective symptomatic treatment option in their patients with nystagmus, although the type of nystagmus was not detailed [42].

### 4.5. Tanganil

The amino acid acetyl-DL-leucine (Tanganil) has been sold over the counter in France for over 65 years and has recently re-emerged as a therapeutic for disorders of the cerebellum [43]. Animal studies have shown Tanganil to increase metabolic activity in the flocculus and stabilize errant membrane potentials in cerebellar neurons that participate in the central vestibular and ocular-motor system [44,45]. All three patients in a trial of Tanganil for Ataxia Telangiectasia who had DBN demonstrated reduction in SPV after 1 month on treatment [46]. Another study of patients with various degenerative cerebellar ataxias showed success in treatment of DBN in the two patients who possessed it [47]. Other reports not explicitly measuring DBN describe improvements in a wide variety of ocular-motor disorders and cerebellar syndromes, but a negative RCT has shown need for further refinement of patient selection [46,47,48,49,50]. Further still, the side effect profile of Tanganil appears safe and tolerable, yielding promise for the future [50].

## 5. Non-Pharmacologic Interventions for Downbeat Nystagmus

Despite the encouraging results of pharmacologic interventions, it is crucially important to understand the limitations of such an approach. It is not uncommon to experience a medication contraindication in patients with CNS disease producing DBN. For example, certain patients may be prone to seizures, like our patient with MOGAD. Patients with Spinocerebellar Ataxia 27b (SCA27b) and DBN may have renal impairment that precludes certain medications.

Although some patients may show objective measures of slow phase velocity improvement in their DBN, others did not report a concomitant relief from their symptoms, such as dizziness or oscillopsia [28]. This discrepancy poses both mechanistic and therapeutic challenges for the treating clinician. The disability incurred by patients with DBN can span multiple modalities, including gait instability, decreases in visual acuity, instability of the visual environment, and disequilibrium or vertigo. The cumulative effect of these visual and balance symptoms can lead to chronic imbalance and a substantial reduction in daily functioning and quality of life. Falls remain a major risk in the DBN population and cause morbidity and mortality in many patients.

A comprehensive rehabilitation program must be prepared to accommodate a variety of symptomatic phenotypes in the DBN syndrome (Table 2).

### 5.1. Vestibular Rehabilitation Therapy

Vestibular Rehabilitation Therapy (VRT) is a highly individualized, exercise-based treatment program fundamentally designed to promote vestibular compensation mechanisms: adaptation, substitution, and habituation [51,52]. There is robust data supporting VRT for peripheral vestibular disorders; however, evidence supporting VRT for central disorders remains less represented in the literature, and only one case report specifically addresses rehabilitation in DBN [53,54,55,56]. The cerebellar dysfunction underlying most DBN cases fundamentally impairs the adaptive mechanisms that VRT typically exploits. Traditional VRT protocols face unique challenges when applied to DBN due to its central origin, where outcomes are less systematically studied [51,53]. Gaze stabilization exercises must account for the gravity-dependent worsening characteristic of DBN or the potential effects of convergence on nystagmus intensity. Balance training protocols require modification to specifically target the pronounced anteroposterior instability that characterizes the DBN syndrome, as documented by Schniepp et al. in their analysis of gait disorders in DBN patients [57]. Proprioceptive training exercises, including weight-shifting on compliant surfaces and dual-task activities that challenge somatosensory integration, complement traditional VRT by enhancing postural control when visual input is compromised by oscillopsia [58,59,60]. Further rehabilitation research should explore whether the varying phenotypes of DBN (gravity-dependent, gravity-independent, etc.) may respond better to specific rehabilitative regimens that capitalize on intact cerebellar circuitry.

### 5.2. Neurovisual Training and Biofeedback

Biofeedback using neurovisual and auditory cueing, emphasizing voluntary control, demonstrates rehabilitative promise in congenital nystagmus, but the acquired nature and pathophysiology of DBN likely requires different paradigms [61,62]

A pilot study by Theil et al. employed real-time computer-based visual feedback using the EyeSeeCam system in 10 patients with DBN [63]. This investigation demonstrated improved visual acuity under dynamic conditions, suggesting that visual feedback mechanisms may offer advantages over purely auditory approaches. A case report demonstrated improvement in VOG-measured fixation stability and overall physical performance undergoing an intensive personalized audiovisual rehabilitation program that employed visual targets synchronized with auditory cues, delivered in 30-min sessions three times weekly over 12 weeks [64]. Electro-motor devices with ocular-motor quantification input to real-time viewing scene adjustment have shown proof of concept, but remain relegated to the research setting [65].

### 5.3. Prism Compensation

Prisms offer immediate, non-invasive symptom relief by exploiting the unique physiological characteristics of DBN. Base-out prisms leverage the convergence-dampening phenomenon observed in many DBN patients; by shifting the visual image outwards, they force the eyes to converge, thus causing a reduction in DBN. Lavin et al. first reported improvement in a patient with nutritional deficiency-induced DBN using base-out prisms [66]. The typical prescription employs seven diopter base-out prisms combined with −1.00 sphere overcorrection to induce convergence. This approach proves most effective in patients whose DBN demonstrably dampens with convergence effort, though individual responses vary considerably. In gaze-evoked DBN, base-down yoked prisms represent an alternative optical strategy that shifts the entire visual field upward, thereby minimizing the downgaze-induced worsening characteristic of DBN [67].

### 5.4. Surgical Interventions

While detailed discussion exceeds this review’s scope, surgical options for severe, treatment-refractory DBN merit brief mention. Extraocular muscle procedures aimed at dampening nystagmus or shifting null positions have shown promising outcomes in small case reports and series, but no RCTs have been performed compared against medical treatment [68,69,70]. Cerebellar decompression may benefit patients with Chiari malformation-associated DBN, though nystagmus improvement often lags behind other neurological symptoms [71,72].

### 5.5. Integrated Treatment Paradigms

Although high-quality comparative effectiveness studies are lacking, the available literature—consisting primarily of case series, small cohort studies, and clinical experience—suggests that multimodal treatment approaches combining pharmacotherapy with non-pharmacological interventions may offer advantages over monotherapy [3,28,42,56]. When tolerated and available, 4-aminopyridine remains the best-studied pharmacologic treatment for DBN, while optical interventions can provide immediate functional improvement for daily activities. Vestibular rehabilitation and biofeedback may offer additional benefit through promotion of long-term adaptive mechanisms, though their specific efficacy in DBN requires systematic investigation.

## 6. Limitations

This report has several limitations inherent to single-case presentations. The therapeutic response to baclofen observed in our patient cannot be generalized to all MOGAD patients with DBN, as individual responses may vary based on underlying pathophysiology and disease severity. Additionally, the relatively short follow-up period of 8 weeks limits our ability to assess long-term outcomes and potential late complications. The literature review, while comprehensive, is constrained by the paucity of high-quality randomized controlled trials specifically addressing DBN in inflammatory CNS disorders, with most evidence derived from small case series and heterogeneous patient populations. Furthermore, the inability to use 4-aminopyridine due to the patient’s seizure history prevented comparison with the current gold-standard pharmacotherapy for DBN. Future prospective studies with larger cohorts of MOGAD patients presenting with DBN are needed to establish evidence-based treatment algorithms and identify predictors of therapeutic response.

## 7. Conclusions

Critical research priorities for the treatment of DBN include the validation of specific VRT protocols through randomized controlled trials, establishment of standardized outcome measures beyond slow-phase velocity for non-pharmacological interventions, long-term follow-up studies to assess sustained benefits, and refinement of biofeedback protocols. The heterogeneous nature of DBN necessitates individualized treatment approaches, yet evidence-based guidelines remain elusive due to few high-quality studies.

Non-pharmacological interventions offer valuable alternatives and adjuncts to 4 aminopyridine therapy in DBN management. While evidentiary quality remains limited compared to pharmacological trials, optical interventions demonstrate immediate efficacy for symptom relief without potential side effects, and emerging rehabilitation technologies show promise for sustained improvement. Future research must prioritize the development of DBN-specific protocols with rigorous methodology to establish evidence-based guidelines for this disabling condition. Until such evidence emerges, clinicians must rely on careful patient selection, realistic expectation setting, and empirical trials of available interventions guided by individual patient characteristics and treatment goals.

## Figures and Tables

**Table 1:** Summary table which comparatively portrays the relevant agents for DBN. (SPV = slow phase velocity, DBN = downbeat nystagmus, CNS = central nervous system, RCT = randomized controlled trial, BID = twice daily, TID = three times daily).

*Comparative Summary of Pharmacologic Agents for Downbeat Nystagmus*			
*Agent*	*Mechanism of Action*	*Efficacy*	*Contraindications*
***4-Aminopyridine (4-AP)*** *Dalfampridine* *AMPYRA^®^*	*• Voltage-gated potassium channel blocker* *• Restores pacemaker potential of inhibitory cerebellar Purkinje cells* *• Prolongs action-potential length* *•Increases hyperpolarization amplitudes*	*• Reduces slow phase velocities* *• Improves visual acuity* *• Improves postural sway* *• Improves gait and stability* *• Multiple RCTs demonstrate efficacy* *• Dosage: 10 mg twice daily*	*• History of seizures* *• Creatinine clearance ≤50 mL/min*
***3,4-Diaminopyridine (3,4-DAP)*** *Amifampridine* *FIRDAPSE^®^*	*• Similar to 4-AP but methylated at third carbon* *• Less lipid soluble* *• Decreased CNS penetration compared to 4-AP*	*• Significant decrease in SPV and symptoms* *• Inferior efficacy compared to 4-AP in head-to-head RCT* *• Still clinically meaningful improvement*	*• May cause seizures (2% rate)* *• Caution with conditions/medications that lower seizure threshold (e.g., intracerebral neoplasms, theophylline)*
***Clonazepam***	*• Benzodiazepine* *• Mechanism not explicitly stated in document*	*• 12/17 patients showed robust response in case series* *• Dosing: 0.5 mg BID, up-titrated to 1 mg BID as tolerated*	*• Sedation* *• Potential for dependence* *• Controlled substance regulation*
***Baclofen***	*• Generalized restoration of GABAergic inhibition by flocculo-nodular structures onto anterior semicircular canal projections to vestibular nuclei*	*• Improved slow phase velocities* *• Improved subjective vertiginous symptoms* *• Not all patients respond* *• Starting dose: 5 mg TID*	*• Caution in patients already taking other sedating medications given sedating properties*
***Gabapentin***	*• Mechanism not specified for DBN*	*• Minimal effects in RCT (6 patients)* *• Robust response for pendular nystagmus* *• UK survey reports effectiveness (nystagmus type not detailed)*	*• Consider dose-adjustment in patients with renal disease* *• Caution in patients already taking other sedating medications given sedating properties*
***Tanganil*** *Acetyl-DL-leucine*	*• Stabilizes errant membrane potentials in cerebellar neurons* *• Increases metabolic activity in the flocculus*	*• Reduction in SPV after 1 month* *• Success in degenerative cerebellar ataxias with DBN* *• Mixed results (negative RCT suggests need for patient-selection refinement)* *• Safe and tolerable side effect profile*	*• Not available in the United States currently*

**Table 2:** Summary table which comparatively portrays the relevant non-pharmacologic interventions for DBN. (VOG = video-oculography, DBN = downbeat nystagmus, RCT = randomized controlled trial, VRT = vestibular rehabilitation therapy).

*Non-Pharmacological Interventions for Downbeat Nystagmus: Summary of Review Findings*			
*Intervention*	*Mechanism/Approach*	*Evidence/Results*	*Limitations/Considerations*
***Vestibular Rehabilitation Therapy (VRT)***	*• Exercise-based program promoting adaptation, substitution, and habituation* *• Modified gaze stabilization exercises* *• Balance training targeting anteroposterior instability* *• Proprioceptive training on compliant surfaces*	*• Robust data for peripheral disorders* *• Limited evidence for central disorders* *• Only one case report specifically for DBN* *• Schniepp* et al. *documented gait disorder patterns*	*• Cerebellar dysfunction impairs adaptive mechanisms VRT exploits* *• Must account for gravity-dependent worsening* *• Outcomes less systematically studied for central origins*
***Neurovisual Training and Biofeedback***	*• Real-time visual/auditory feedback* *• EyeSeeCam system for computer-based feedback* *• Visual targets synchronized with auditory cues* *• Electro-motor devices with ocular quantification*	*• Theil* et al. *pilot study (n = 10): improved visual acuity under dynamic conditions* *• Case report: improved VOG-measured fixation stability* *• 30-min sessions 3×/week for 12 weeks showed benefit*	*• Acquired DBN requires different paradigms than congenital* *• Advanced systems remain in research settings* *• Limited to small studies and case reports*
***Prism Compensation***	*• Base-out prisms: exploit convergence-dampening* *• Typical: 7 diopter base-out +−1.00 D sphere* *• Base-down yoked prisms: shift visual field upward for gaze-evoked DBN*	*• Lavin et al. first reported improvement* *• Most effective when DBN dampens with convergence* *• Immediate, non-invasive symptom relief*	*• Individual responses vary considerably* *• Effectiveness depends on convergence response* *• Limited to specific DBN phenotypes*
***Surgical Interventions***	*• Extraocular muscle procedures* *• Null position shifting* *• Cerebellar decompression (for Chiari malformation)*	*• Promising outcomes in small case series* *• May benefit Chiari-associated DBN*	*• No RCTs comparing to medical treatment* *• Nystagmus improvement often lags behind other symptoms* *• Reserved for severe, treatment-refractory cases*
***Integrated Treatment Paradigms***	*• Multimodal approaches combining pharmacotherapy with non-pharmacological interventions*	*• Literature suggests advantages over monotherapy* *• 4-aminopyridine + optical interventions common* *• May promote long-term adaptation*	*• High-quality comparative effectiveness studies lacking* *• Evidence primarily from case series and clinical experience* *• Specific efficacy requires systematic investigation*

## Data Availability

No new data were created or analyzed in this study. Data sharing is not applicable to this article.

## Supplementary Materials

The following supporting information can be downloaded at https://www.mdpi.com/article/10.3390/brainsci15080859/s1, File S1: Completed CARE checklist for case report guidelines; Video S1: Demonstrating the nystagmus of the presented case.

## References

[1] Wagner J.N., Glaser M., Brandt T., Strupp M. (2008). Downbeat nystagmus: Aetiology and comorbidity in 117 patients. J. Neurol. Neurosurg. Psychiatry.

[2] Zhang S., Lang Y., Wang W., Wu Y., Yan S., Zhang T., Li D., Liu S., Hao Y., Yang X. (2024). Analysis of etiology and clinical features of spontaneous downbeat nystagmus: A retrospective study. Front. Neurol..

[3] Marcelli V., Giannoni B., Volpe G., Faralli M., Fetoni A.R., Pettorossi V.E. (2024). Downbeat nystagmus: A clinical and pathophysiological review. Front. Neurol..

[4] Lee S.-U., Kim H.-J., Choi J.-Y., Kim J.-S. (2020). Ictal downbeat nystagmus in Ménière disease: A cross-sectional study. Neurology.

[5] Misale P., Hassannia F., Dabiri S., Brandstaetter T., Rutka J. (2021). Post-traumatic peripheral vestibular disorders (excluding positional vertigo) in workers following head injury. Sci. Rep..

[6] Califano L., Salafia F., Mazzone S., Melillo M.G., Califano M. (2014). Anterior canal BPPV and apogeotropic posterior canal BPPV: Two rare forms of vertical canalolithiasis. Acta Otorhinolaryngol. Ital..

[7] Leigh R.J., Zee D.S. (2015). The Neurology of Eye Movements.

[8] Matsuo V., Cohen B. (1984). Vertical optokinetic nystagmus and vestibular nystagmus in the monkey: Up-down asymmetry and effects of gravity. Exp. Brain Res..

[9] Collewijn H. (1972). An analog model of the rabbit’s optokinetic system. Brain Res..

[10] Fernández C., Goldberg J.M. (1976). Physiology of peripheral neurons innervating otolith organs of the squirrel monkey. I. Response to static tilts and to long-duration centrifugal force. J. Neurophysiol..

[11] Merfeld D.M. (1995). Modeling the vestibulo-ocular reflex of the squirrel monkey during eccentric rotation and roll tilt. Exp. Brain Res..

[12] Laurens J., Angelaki D.E. (2011). The functional significance of velocity storage and its dependence on gravity. Exp. Brain Res..

[13] Cohen B., John P., Yakushin S.B., Buettner-Ennever J., Raphan T. (2002). The nodulus and uvula: Source of cerebellar control of spatial orientation of the angular vestibulo-ocular reflex. Ann. N. Y. Acad. Sci..

[14] Helmchen C., Gottschalk S., Sander T., Trillenberg P., Rambold H., Sprenger A. (2007). Beneficial effects of 3,4-diaminopyridine on positioning downbeat nystagmus in a circumscribed uvulo-nodular lesion. J. Neurol..

[15] Walker M.F., Tian J., Shan X., Tamargo R.J., Ying H., Zee D.S. (2008). Lesions of the cerebellar nodulus and uvula impair downward pursuit. J. Neurophysiol..

[16] Crawford J.D., Cadera W., Vilis T. (1991). Generation of Torsional and Vertical Eye Position Signals by the Interstitial Nucleus of Cajal. Science.

[17] Robinson D.A. (2022). Neurophysiology, pathology and models of rapid eye movements. Prog. Brain Res..

[18] Strupp M., Kremmyda O., Adamczyk C., Böttcher N., Muth C., Yip C.W., Bremova T. (2014). Central ocular motor disorders, including gaze palsy and nystagmus. J. Neurol..

[19] Nakamagoe K., Shimizu K., Koganezawa T., Tamaoka A. (2012). Downbeat nystagmus due to a paramedian medullary lesion. J. Clin. Neurosci..

[20] Helmchen C., Rambold H., Fuhry L., Büttner U. (1998). Deficits in vertical and torsional eye movements after uni- and bilateral muscimol inactivation of the interstitial nucleus of Cajal of the alert monkey. Exp. Brain Res..

[21] Claassen J., Feil K., Bardins S., Teufel J., Spiegel R., Kalla R., Schneider E., Jahn K., Schniepp R., Strupp M. (2013). Dalfampridine in patients with downbeat nystagmus--an observational study. J. Neurol..

[22] Alviña K., Khodakhah K. (2010). The therapeutic mode of action of 4-aminopyridine in cerebellar ataxia. J. Neurosci..

[23] Shaikh A.G. (2013). Does 4-aminopyridine ‘beat’ downbeat nystagmus?. J. Neurol. Neurosurg. Psychiatry.

[24] Kalla R., Spiegel R., Claassen J., Bardins S., Hahn A., Schneider E., Rettinger N., Glasauer S., Brandt T., Strupp M. (2011). Comparison of 10-mg doses of 4-aminopyridine and 3,4-diaminopyridine for the treatment of downbeat nystagmus. J. Neuro-Ophthalmol..

[25] Kalla R., Glasauer S., Büttner U., Brandt T., Strupp M. (2007). 4-aminopyridine restores vertical and horizontal neural integrator function in downbeat nystagmus. Brain J. Neurol..

[26] Kalla R., Glasauer S., Schautzer F., Lehnen N., Büttner U., Strupp M., Brandt T. (2004). 4-aminopyridine improves downbeat nystagmus, smooth pursuit, and VOR gain. Neurology.

[27] Kremmyda O., Zwergal A., la Fougère C., Brandt T., Jahn K., Strupp M. (2013). 4-Aminopyridine suppresses positional nystagmus caused by cerebellar vermis lesion. J. Neurol..

[28] Strupp M., Teufel J., Zwergal A., Schniepp R., Khodakhah K., Feil K. (2017). Aminopyridines for the treatment of neurologic disorders. Neurol. Clin. Pract..

[29] Inc. Acorda Therapeutics (2010). Ampyra (Package Insert).

[30] Strupp M., SchülEr O., Krafczyk S., Jahn K., Schautzer F., BütTner U., Brandt T. (2003). Treatment of downbeat nystagmus with 3,4-diaminopyridine: A placebo-controlled study. Neurology.

[31] Tsunemi T., Ishikawa K., Tsukui K., Sumi T., Kitamura K., Mizusawa H. (2010). The effect of 3,4-diaminopyridine on the patients with hereditary pure cerebellar ataxia. J. Neurol. Sci..

[32] Lemeignan M., Millart H., Lamiable D., Molgo J., Lechat P. (1984). Evaluation of 4-aminopyridine and 3,4-diaminopyridine penetrability into cerebrospinal fluid in anesthetized rats. Brain Res..

[33] Catalyst Pharmaceuticals (2024). Firdapse (Package Insert). https://www.accessdata.fda.gov/drugsatfda_docs/label/2024/208078s012lbl.pdf.

[34] Wirtz P.W., Titulaer M.J., van Gerven J.M., Verschuuren J.J. (2010). 3,4-diaminopyridine for the treatment of Lambert-Eaton myasthenic syndrome. Expert Rev. Clin. Immunol..

[35] Sanders D.B. (1998). 3,4-Diaminopyridine (DAP) in the treatment of Lambert-Eaton myasthenic syndrome (LEMS). Ann. N. Y. Acad. Sci..

[36] McEvoy K.M., Windebank A.J., Daube J.R., Low P.A. (1989). 3,4-Diaminopyridine in the treatment of Lambert-Eaton myasthenic syndrome. N. Engl. J. Med..

[37] Young Y.H., Huang T.W. (2001). Role of clonazepam in the treatment of idiopathic downbeat nystagmus. Laryngoscope.

[38] Currie J.N., Matsuo V. (1986). The use of clonazepam in the treatment of nystagmus-induced oscillopsia. Ophthalmology.

[39] Yun S.-Y., Lee J.-H., Kim H.-J., Choi J.-Y., Kim J.-S. (2024). Effects of Baclofen on Central Paroxysmal Positional Downbeat Nystagmus. Cerebellum.

[40] Dieterich M., Straube A., Brandt T., Paulus W., Büttner U. (1991). The effects of baclofen and cholinergic drugs on upbeat and downbeat nystagmus. J. Neurol. Neurosurg. Psychiatry.

[41] Averbuch-Heller L., Tusa R.J., Fuhry L., Rottach K.G., Ganser G.L., Heide W., Büttner U., Leigh R.J. (1997). A double-blind controlled study of gabapentin and baclofen as treatment for acquired nystagmus. Ann. Neurol..

[42] Choudhuri I., Sarvananthan N., Gottlob I. (2007). Survey of management of acquired nystagmus in the United Kingdom. Eye.

[43] Vlček P., Horáček J., Grünerová-Lippertová M., Brunovský M. (2025). Therapeutic potential of acetyl-DL-leucine and its L-enantiomer in posterior fossa syndrome: Mechanistic insights. Drug Discov. Today.

[44] Vibert N., Vidal P.P. (2001). In vitro effects of acetyl-DL-leucine (tanganil) on central vestibular neurons and vestibulo-ocular networks of the guinea-pig. Eur. J. Neurosci..

[45] Günther L., Beck R., Xiong G., Potschka H., Jahn K., Bartenstein P., Brandt T., Dutia M., Dieterich M., Strupp M. (2015). N-acetyl-L-leucine accelerates vestibular compensation after unilateral labyrinthectomy by action in the cerebellum and thalamus. PLoS ONE.

[46] Brueggemann A., Bicvic A., Goeldlin M., Kalla R., Kerkeni H., Mantokoudis G., Abegg M., Kolníková M., Mohaupt M., Bremova-Ertl T. (2022). Effects of Acetyl-DL-Leucine on Ataxia and Downbeat-Nystagmus in Six Patients With Ataxia Telangiectasia. J. Child Neurol..

[47] Strupp M., Teufel J., Habs M., Feuerecker R., Muth C., van de Warrenburg B.P., Klopstock T., Feil K. (2013). Effects of acetyl-DL-leucine in patients with cerebellar ataxia: A case series. J. Neurol..

[48] Kremmyda O., Feil K., Bardins S., Strupp M. (2023). Acetyl-DL-leucine in combination with memantine improves acquired pendular nystagmus caused by multiple sclerosis: A case report. J. Neurol..

[49] Bremova-Ertl T., Ramaswami U., Brands M., Foltan T., Gautschi M., Gissen P., Gowing F., Hahn A., Jones S., Kay R. (2024). Trial of *N* -Acetyl-l-Leucine in Niemann–Pick Disease Type C. N. Engl. J. Med..

[50] Feil K., Adrion C., Boesch S., Doss S., Giordano I., Hengel H., Jacobi H., Klockgether T., Nachbauer W., Schöls L. (2021). Safety and Efficacy of Acetyl-DL-Leucine in Certain Types of Cerebellar Ataxia: The ALCAT Randomized Clinical Crossover Trial. JAMA Netw. Open..

[51] Shepard N.T., Telian S.A., Smith-Wheelock M. (1990). Habituation and balance retraining therapy. A retrospective review. Neurol. Clin..

[52] Han B.I., Song H.S., Kim J.S. (2011). Vestibular rehabilitation therapy: Review of indications, mechanisms, and key exercises. J. Clin. Neurol. Seoul Korea.

[53] McDonnell M.N., Hillier S.L. (2015). Vestibular rehabilitation for unilateral peripheral vestibular dysfunction. Cochrane Database Syst. Rev..

[54] Tramontano M., Russo V., Spitoni G.F., Ciancarelli I., Paolucci S., Manzari L., Morone G. (2021). Efficacy of Vestibular Rehabilitation in Patients With Neurologic Disorders: A Systematic Review. Arch. Phys. Med. Rehabil..

[55] Synofzik M., Ilg W. (2014). Motor training in degenerative spinocerebellar disease: Ataxia-specific improvements by intensive physiotherapy and exergames. BioMed Res. Int..

[56] Cornforth E., Schmahmann J.D. (2025). Physical Therapy and Aminopyridine for Downbeat Nystagmus Syndrome: A Case Report. J. Neurol. Phys. Ther. JNPT.

[57] Schniepp R., Wuehr M., Huth S., Pradhan C., Schlick C., Brandt T., Jahn K., Thurtell M. (2014). The gait disorder in downbeat nystagmus syndrome. PLoS ONE.

[58] Geisinger D., Elyoseph Z., Zaltzman R., Mintz M., Gordon C.R. (2024). Functional impact of bilateral vestibular loss and the unexplained complaint of oscillopsia. Front. Neurol..

[59] Sparrer I., Dinh T.A.D., Ilgner J., Westhofen M. (2013). Vestibular rehabilitation using the Nintendo^®^ Wii Balance Board—A user-friendly alternative for central nervous compensation. Acta Otolaryngol..

[60] Hall C.D., Herdman S.J.P., Whitney S.L.D., Anson E.R., Carender W.J.P., Hoppes C.W.P., Cass S.P., Christy J.B., Cohen H.S.O., Fife T.D.M. (2022). Vestibular Rehabilitation for Peripheral Vestibular Hypofunction: An Updated Clinical Practice Guideline From the Academy of Neurologic Physical Therapy of the American Physical Therapy Association. J. Neurol. Phys. Ther. JNPT.

[61] Abadi R.V., Carden D., Simpson J. (1980). A new treatment for congenital nystagmus. Br. J. Ophthalmol..

[62] Ciuffreda K.J., Goldrich S.G., Neary C. (1982). Use of eye movement auditory biofeedback in the control of nystagmus. Am. J. Optom. Physiol. Opt..

[63] Teufel J., Bardins S., Spiegel R., Kremmyda O., Schneider E., Strupp M., Kalla R. (2016). Real-time computer-based visual feedback improves visual acuity in downbeat nystagmus—A pilot study. J. Neuroeng. Rehabil..

[64] Antognetti D., Maggiani L., Gabbrielli E., Allegrini L., Dalise S., Chisari C. (2024). Neurovisual Training With Acoustic Feedback: An Innovative Approach for Nystagmus Rehabilitation. Arch. Rehabil. Res. Clin. Transl..

[65] Smith R.M., Oommen B.S., Stahl J.S. (2004). Image-shifting optics for a nystagmus treatment device. J. Rehabil. Res. Dev..

[66] Lavin P.J., Traccis S., Dell’Osso L.F., Abel L.A., Ellenberger C. (1983). Downbeat nystagmus with a pseudocycloid waveform: Improvement with base-out prisms. Ann. Neurol..

[67] Hertle R.W. (2000). Examination and refractive management of patients with nystagmus. Surv. Ophthalmol..

[68] Wang Z.I., Dell’Osso L.F., Tomsak R.L., Jacobs J.B. (2007). Combining recessions (nystagmus and strabismus) with tenotomy improved visual function and decreased oscillopsia and diplopia in acquired downbeat nystagmus and in horizontal infantile nystagmus syndrome. J. AAPOS.

[69] Depalo C., Hertle R.W., Yang D. (2003). Eight eye muscle surgical treatment in a patient with acquired nystagmus and strabismus: A case report. Binocul. Vis. Strabismus Q..

[70] Hertle R.W., Ahmad A. (2019). Clinical and electrophysiological results of eye muscle surgery in 17 patients with downbeat nystagmus. Indian J. Ophthalmol..

[71] Spooner J.W., Baloh R.W. (1981). Arnold-Chiari malformation: Improvement in eye movements after surgical treatment. Brain J. Neurol..

[72] Denion E., Defoort-Dhellemmes S., Arndt C.-F., Bouvet-Drumare I., Beaussart K., Hache J.-C., Dhellemmes P. (2001). Improvement of downbeat nystagmus after suboccipital decompression for Chiari I malformation. Neuro-Ophthalmology.

